# Nut Intake, Functional Limitations, and Quality of Life in Older Adults: Findings From NHANES 2003–2012

**DOI:** 10.1002/jcsm.70022

**Published:** 2025-07-31

**Authors:** Ilili Feyesa, Elena S. George, David Scott, Gavin Abbott, Jeew Hettiarachchi, Robin M. Daly, Jack Dalla Via, Ekavi N. Georgousopoulou, Sze‐Yen Tan

**Affiliations:** ^1^ Institute for Physical Activity and Nutrition (IPAN), School of Exercise and Nutrition Sciences Deakin University Geelong VIC Australia; ^2^ School of Clinical Sciences at Monash Health Monash University Clayton VIC Australia; ^3^ Nutrition and Health Innovation Research Institute, School of Medical and Health Sciences Edith Cowan University Joondalup WA Australia; ^4^ Discipline of Nutrition and Dietetics University of Canberra Canberra Australia; ^5^ School of Medicine Sydney University of Notre Dame Australia Sydney Australia

**Keywords:** functional limitation, nuts, older adult, quality of life

## Abstract

**Background:**

Increased nut consumption has positive effects on physical and cognitive function, but whether these translate into lower functional limitations in older adults is unknown. The aim of this study was to investigate the association between nut intake, functional limitations, the severity of these limitations and health‐related quality of life (HRQOL) in older adults.

**Methods:**

This cross‐sectional study included data from 5807 adults (53.4% female) aged 60 years and older who participated in the US National Health and Nutrition Examination Survey (NHANES) between 2003 and 2012. Nut intake was assessed using two 24‐h dietary recalls. Functional limitations were assessed using the NHANES Physical Functioning Questionnaire. Quality of life was evaluated using the four‐question HRQOL‐4 questionnaire. Negative binomial regression, linear regression and logistic regression analyses were used to examine associations between nut intake and both functional limitations and quality of life.

**Results:**

Nut consumption (> 0 g/day) was associated with significantly fewer functional limitations (β = −0.12; 95% CI: −0.24, −0.01; *p* = 0.048) and lower severity of these limitations (β = −0.67; 95% CI −1.11, −0.23; *p* = 0.004) compared to no consumption. Compared to nonconsumers, nut consumers (> 0 g/day) also had lower odds of reporting poor physical health (OR = 0.81; 95% CI 0.68, 0.98; *p* = 0.027) and activity limitations (OR = 0.72; 95% CI 0.55, 0.95; *p* = 0.023). When participants were categorised by intake level (nonconsumers, < 6.9 g/day, and ≥ 6.9 g/day), consuming ≥ 6.9 g/day was associated with fewer (β = −0.19; 95% CI: −0.32, −0.06; *p* = 0.004) and lower severity of functional limitations (β = −0.84; 95% CI: −1.30, −0.37; *p* = 0.001) compared to no consumption. Intake ≥ 6.9 g/day was also associated with lower odds of poor general health (OR = 0.76; 95% CI: 0.60, 0.906; *p* = 0.023), poor physical health (OR = 0.74; 95% CI: 0.57, 0.97; *p* = 0.027) and limitations in daily activities (OR = 0.61; 95% CI: 0.46, 0.82; *p* = 0.001).

**Conclusions:**

Consumption of nuts, particularly at above‐median levels, in older adults may be linked with experiencing fewer functional limitations, lower severity of these limitations and fewer general and physical unhealthy and inactive days.

## 1 Introduction

Ageing is associated with functional limitations [1] that can result from physical, cognitive and social impairments that affect an individual's ability to independently carry out activities of daily living (ADLs), including basic activities such as bathing, dressing, eating and toileting, as well as instrumental activities encompassing more complex tasks like managing finances and household chores [2, 3]. These limitations can negatively impact older adults' independence, quality of life and disease risk and progression [1, 4]. However, there is significant variability in how ageing affects health in later life [5, 6] with evidence showing that modifiable behavioural factors, such as diet, can play a role in the development of functional limitations.

Growing evidence suggests that adherence to a diet high in nuts, whole grains, vegetables and fruits, a moderate intake of alcohol, dairy products and olive oil; and a low intake of meat (e.g., the Mediterranean diet) is positively associated with mobility and general physical functioning [7, 8]. Consuming adequate nutrients that are essential for good health, such as protein, unsaturated fats and various vitamins and minerals, has also been recommended to prevent age‐related functional declines [9, 10]. Nuts are nutrient‐dense foods that are rich in plant protein, phytochemicals, vitamins (folate, niacin and vitamin E), minerals (selenium, magnesium, calcium and potassium), fibre and mono‐ and polyunsaturated fats. These nutrients have been shown to play a role in reducing chronic systemic inflammation and oxidative stress, which have been linked to functional declines with ageing and disease [11].

Few studies have investigated the association between nut consumption and functional limitation in older adults. These studies [12, 13] reported a positive association between nut consumption and a lower risk of functional limitations, particularly with activities such as bending or kneeling, carrying shopping bags, climbing stairs or walking several blocks [12], as well as basic activities such as bathing, dressing, using the toilet, moving from a chair or bed, walking short distances and eating [13]. However, these previous studies concentrated on a very limited range of function measures (i.e., ADLs) and have not examined the severity of functional limitations, which enriched the information on the number of limitations. Therefore, the current study aims to examine whether nut consumption is associated with functional limitation across a range of activities, the severity of these limitations and health‐related quality of life (HRQOL) in adults aged 60 years and older.

## 2 Methods

### 2.1 Study Population

**FIGURE 1:**
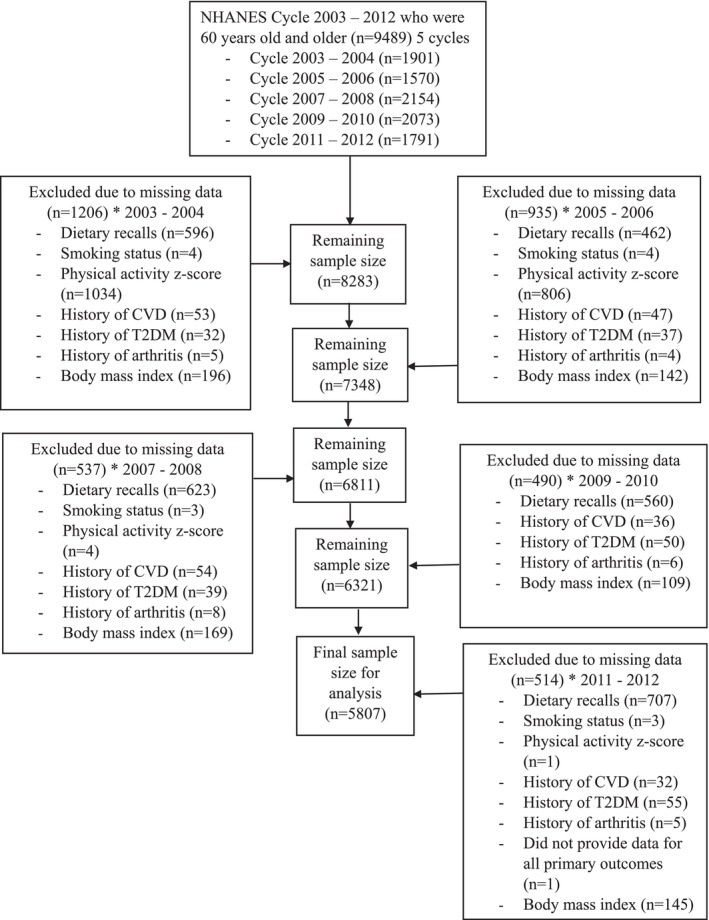
Participant exclusion flow chart for all outcomes* Numbers of individual variables do not add up to a total number of excluded participants as some could have missing data for more than one variable.

This **cross‐sectional** study included older adults aged 60 years and over who participated in the National Health and Nutrition Examination Survey (NHANES) from 2003 to 2012 (5 cycles) based on the availability of exposure and outcome data required for our analyses. NHANES was conducted by the National Centre for Health Statistics (NCHS) and Centres for Disease Control and Prevention (CDC) agency and employs a complex, multistage probability sampling procedure to estimate the health and nutritional status of noninstitutionalised residents in the United States. The survey protocols for NHANES 2003–2012 (Protocol #98‐12, Protocol #2005‐06 and Protocol #2011–17) were approved by the NCHS Research Ethics Review Board, and all participants gave informed consent [14]. As part of NHANES, participants provided information regarding demographics, socioeconomic status, diet and health in an interview, underwent medical and physiological examinations and provided fasting blood samples. A total of 9489 participants aged 60 years and over were initially identified for this study. Participants were included if they had completed two reliable 24‐h recalls and had complete data on relevant covariates (age, sex, ethnicity, education status, body mass index (BMI), history of arthritis, diabetes, prior cardiovascular event, smoking status and physical activity). Thus, our analyses included a final sample of 5807 older adults who met all inclusion criteria and had complete data for analysis as shown in Figure 1.

### 2.2 Dietary Assessment, Nut Intake and Diet Quality

NHANES collected information on dietary intake using a multiple‐pass 24‐h recall method on weekdays and weekends, administered by trained interviewers using the US Department of Agriculture's (USDA) Automated Multiple‐Pass Method (AMPM). The AMPM is a commonly used dietary assessment method in national nutrition surveys [15]. The 5‐step AMPM process includes (1) participants listing all foods and beverages, (2) the interviewer prompting the participants about foods that they might have forgotten, (3) eating time and occasion documented, (4) additional details obtained (e.g., preparation methods, portion sizes and additions such as sugar and spread) and (5) a final probe that ensured no missing data on dietary intake. The accuracy of this method has been demonstrated in a previous study [16]. To ensure the accuracy of the dietary data, a subset of survey participants completed a second 24‐h recall. Nutrient analysis was conducted on the recalls using the USDA Food Composition Databases to determine total nutrient intake.

Since nuts are an occasional food, we calculated the mean intake of nuts, including ‘almonds, almond butter, Brazil nuts, cashews, cashew butter, hazelnuts, macadamias, pecans, pine nuts, pistachios, walnuts, peanuts, and peanut butter’, from two 24‐h dietary recalls, as per our previous studies [17, 18]. To ensure nut intake was captured more accurately, we considered nuts eaten on their own and disaggregated nut intake from recipes and mixed dishes using the Food Commodity Intake Database (FCID) to capture an accurate estimate of the total nut intake from all food sources. The FCID is a comprehensive food composition database, which disaggregates NHANES foods into nearly 500 highly differentiated ingredients (e.g., all types of nuts) and has been used to evaluate dietary intake, chemical exposure, environmental impacts, agricultural resource use and food expenditures [19]. This method allows the quantification of the amount of peanut butter in a peanut butter and jelly sandwich [17].

The diet quality of the participants was assessed by the Healthy Eating Index‐2020 (HEI‐2020) [20] calculated using the recently developed ‘dietaryindex’, an R package that provides user‐friendly and streamlined methods to standardise the compilation of dietary intake data into index‐based dietary patterns [21]. The HEI‐2020 included nine components related to adequacy—‘total fruit, whole fruit, total vegetables, greens and beans, whole grains, dairy, total protein foods, seafood and plant proteins and fatty acids’ and four components related to moderation—‘refined grains, sodium, added sugars and saturated fat’. A maximum of 5 points were awarded to ‘total fruit’, ‘whole fruits’, ‘total vegetables’, ‘greens and beans’, ‘total protein foods’ and ‘seafood and plant proteins’; and a maximum of 10 points for ‘whole grains’, ‘dairy’, ‘fatty acids’, ‘refined grains’, ‘sodium’, ‘added sugars’ and ‘saturated fats’. The total score ranges from 0 to 100, with a higher score indicating a greater intake of adequacy components and a lower intake of moderation components [20]. HEI‐2020 scores were calculated for both 24‐h dietary recalls, and the average scores are used for the current analyses.

### 2.3 Functional Limitations

The primary outcome of this analysis was functional limitation evaluated using the NHANES 19‐item validated Physical Functioning Questionnaire [22, 23]. Participants were asked to rate their ability to perform 19 common daily life activities, ranging from *No difficulty* (severity score 0), *Some difficulty* (severity score 1), *Much difficulty* (severity score 2) to *Unable to do* (severity score 3). Participants' responses to the Physical Functioning Questionnaire were scored in two ways: (i) as the total number (score range 0–19) of functional limitations (function limitation was defined as a report of some or much difficulty or unable to do the activity and was assigned a score of 1, while reporting no difficulty was assigned a score of 0) and (ii) as the severity of functional limitation (composite score that combines the number of limitations (0–19) multiplied by the severity of each limitation (0–3)) [24].

### 2.4 Quality of Life

The secondary outcome of this study was HRQOL evaluated using the HRQOL‐4 questionnaire from the NHANES Current Health Questionnaire. HRQOL‐4 is a validated tool [25] endorsed by the US Centers for Disease Control and Prevention as a population surveillance tool to assess individuals' or groups' perceived physical and mental health. It consists of four questions focusing on the participant's general health status (Question 1), number of physically unhealthy days in the 30 days preceding the survey (Question 2), number of mentally unhealthy days in the 30 days preceding the survey (Question 3) and number of days with activity limitations in the 30 days preceding the survey (Question 4). Question 1 predicts mortality and chronic disease conditions, Questions 2 and 3 assess recent physical symptoms and mental or emotional distress, respectively, and Question 4 measures perceived disability and lost productivity [26]. Based on previous studies [27, 28], we dichotomised each HRQOL‐4 component variable into good versus poor categories: General health was classified as good (good, very good or excellent health) versus poor (poor or fair), physical and mental health as good (< 14 days/month) versus poor (≥ 14 days/month) and activity limitation as good (< 14 days/month) versus poor (≤ 14 days/month).

### 2.5 Covariate Assessment

Participants' ethnic group (Mexican American, Non‐Hispanic White, Non‐Hispanic Black, Non‐Hispanic Asian and others), education status (< 11th grade, high school graduate, some college or associates (AA) degree and college graduate or higher) and household size were collected using demographic questionnaires, which were administered by trained interviewers using a Computer‐Assisted Personal Interviewing system. Trained health technicians performed weight and height measurements using standard examination protocols in a Mobile Examination Centre [17]. Height (cm) was measured using a stadiometer, and body weight (kg) using a digital scale, with participants wearing a standard examination gown with a disposable shirt, pants and slippers, with only underwear underneath the gown. BMI was calculated as weight (kg) divided by height (metres) squared.

Self‐reported frequency and duration of leisure‐time physical activity (PA) from the previous week or month were collected from participants using different questionnaires across the 5 cycles of NHANES included in this study. We converted the reported daily leisure‐time PA (total minutes of moderate‐ and vigorous‐intensity PA (as METS· min/day)) into a standardised *z*‐score for each cycle to ensure consistency in the measurement of this outcome during daily leisure‐time PA, as reported elsewhere [29, 30]. This was obtained by multiplying the number of days per week an individual engaged in moderate‐ or vigorous‐intensity activity by the duration (minutes) of PA on each of those days, which was then converted into daily minutes of moderate‐ and vigorous‐intensity PA. The *z*‐score was then calculated using the mean and SD of the total sample for each NHANES cycle.

Participants' smoking status was assessed during the interview through two questions: ‘Have you smoked at least 100 cigarettes in your entire life?’ and ‘Do you now smoke cigarettes?’ Individuals who responded ‘no’ to the first question were considered non‐smokers; those who answered ‘yes’ to the first but ‘not at all’ to the second questions were considered ex‐smokers, and those who answered ‘yes’ to the first questions and ‘every day’ or ‘some days’ to the second question were considered as current smokers.

Participants' history of cardiovascular disease **(**CVD), type 2 diabetes mellitus (T2DM) and arthritis, which may affect functional capacity, was collected through an interview [13]. Participants were considered to have a history of CVD if they had been told that they had angina/angina pectoris, coronary heart disease, stroke, congestive heart failure or heart attack. History of T2DM was based on participants' self‐reported diagnosis of diabetes or those who did not report T2DM diagnosis but had a fasting HbA1c greater than 6.4% [18]. History of arthritis was based on participants' self‐reported diagnosis of arthritis.

### 2.6 Statistical Analysis

All statistical analyses were performed using Stata 18, and statistical significance was set at *p* < 0.05. Ten‐year weights for the 2003–2012 survey period were created by combining 2‐year sample weights from each NHANES cycle [14]. All analyses utilised the 2‐day dietary recall weights. Descriptive statistics were performed using survey‐weighted proportions and means for categorical variables and continuous variables, respectively. The mean difference in general characteristics between non‐nut consumers and nut consumers was tested using an *F*‐test, whereas the differences in proportions were tested using the chi‐square test.

Participants were categorised as nonconsumers (0 g/day) and nut consumers (> 0 g/day) and this variable was analysed as a binary exposure, with the nonconsumer group set as the reference group. To further investigate the potential dose at which the effect occurs, participants were also classified into three groups based on the median nut intake among consumers in the sample (6.9 g/day): nonconsumers, consumers with intake below the median (< 6.9 g/day) and those with intake at or above the median (≥ 6.9 g/day). This categorical exposure variable was analysed using nonconsumers as the reference category. Negative binomial regression models were conducted to estimate associations between nut consumption and the number of functional limitations. Multiple linear regression models were used to investigate associations between nut intake and severity of functional limitation. Binary logistic regression models were employed to examine associations between nut consumption and dichotomised HRQOL outcomes. All regression models were adjusted for age, sex, BMI, ethnicity, household size, education status, smoking status, history of CVD, history of T2DM, history of arthritis, physical activity and diet quality (HEI‐2020). Diet quality was included as a covariate because nut intake has been shown to improve diet quality [20], which in turn may influence the functional capacity and quality of life outcomes of this study. We tested for sex, physical activity and obesity status interactions in the association between nut consumption and the number of functional limitations; however, we found no evidence of significant interactions, and so results for these variables are combined.

## 3 Results

### 3.1 Characteristics of Participants

Table 1 presents the general characteristics of nut consumers and nonconsumers. Nut consumers had a higher education level, were more active and were less likely to be current smokers. They were also less likely to have a history of cardiovascular events and T2DM. Nut consumers had a lower BMI and better diet based on HEI‐2020. The median daily intake of nuts among consumers was 6.9 g/day (IQR: 1.8, 18.3).

**TABLE 1:** Characteristics of older adults aged 60 years and over in the NHANES 2003–2012 based on nut intake categories.

	Nut intake categories		
Characteristics	Nonconsumers (0 g/day) (38.2%)	Consumers (> 0 g/day) (61.8%)	*P*‐value^a^
Mean age in years	69.6 (69.2, 70.1)	69.5 (69.2, 69.9)	0.724
Sex, females (%)	53.4	56.0	0.182
Racial group (%)			< 0.001
Mexican American	5.1	2.5	
Hispanic	5.0	1.9	
Non‐Hispanic White	73.5	86.3	
Non‐Hispanic Black	11.9	5.9	
Other	4.5	3.3	
Educational status (%)			< 0.001
< 11 Grade	29.2	14.9	
High School graduate	27.3	25.2	
Some college or AA degree	24.3	27.7	
College graduate or above	19.2	32.2	
Household size (%)			< 0.001
Lives alone ^j^	24.7	24.0	
Two people in a household	55.3	59.0	
> 2 people in household	20.0	17.0	
Smoking status (%)			< 0.001
Never smoked	47.6	48.9	
Ex‐smoker	38.2	42.6	
Current smoker	14.2	8.6	
Mean physical activity, *z*‐score	−0.30 (−0.33, −0.27)	−0.21 (−0.25, −0.18)	< 0.001
History of T2DM (%)	25.2	19.3	< 0.001
History of CVD (%)	23.2	20.1	0.040
History of arthritis (%)	48.7	49.9	0.479
BMI (kg/m^2^)	29.6 (29.1, 29.9)	28.5 (28.0, 28.9)	< 0.001
Nut intake (g/day)^b^	0	6.9 [1.8, 18.2]	
HEI‐2020	51.5 (50.9, 52.2)	58.2 (57.4, 59.0)	< 0.001

*Note:* All values are weighted mean (95% CI) or weighted % unless otherwise stated.

^a^ *P*‐value for differences between nonconsumers and consumers based on *F*‐test (continuous variable) or design‐based *F*‐test (categorical variables).

^b^ Values are median [IQR‐interquartile range].

Abbreviations: BMI, body mass index; CVD, Cardiovascular Disease; HEI‐2020, Healthy Eating Index 2020 scores; T2DM, Type 2 diabetes.

### 3.2 Nuts and Functional Limitations

Table 2 presents the frequency of functional limitations among nonconsumers and consumers of nuts. A statistically significantly higher proportion of nonconsumers reported limitations in all measures except for using fork, knife and drinking from a cup; dressing yourself; and grasp/holding a small object. The most prevalent limitations across both groups included: stooping, crouching and kneeling; standing up from an armless chair; and walking for a quarter mile.

**TABLE 2:** Proportion of older adults aged 60 years and over who reported functional limitations and poor quality of life according to nut intake categories (nonconsumers vs. consumers) in NHANES 2003–2012.

Functional limitations^a^	Nonconsumers (0 g/day) (38.2%)	Consumers (> 0 g/day) (61.8%)	*P*‐value^b^
Managing money (%)	6.3	3.7	0.001
Walking for a quarter mile (%)	34.8	24.2	< 0.001
Walking up 10 steps (%)	29.3	19.3	< 0.001
Stooping, crouching and kneeling (%)	46.9	41.8	0.004
Lifting or carrying difficulty (%)	21.6	13.9	< 0.001
House chore (%)	20.2	14.8	< 0.001
Preparing meals (%)	7.8	3.8	< 0.001
Walking between rooms on the same floor (%)	7.9	4.8	0.001
Standing up from armless chair (%)	20.4	15.3	0.001
Getting in and out of bed (%)	12.0	8.2	0.002
Using fork, knife and drinking from cup (%)	4.3	3.3	0.084
Dressing yourself (%)	10.0	7.8	0.055
Standing up from armless chair (%)	39.1	33.3	0.004
Sitting for long periods (%)	16.7	13.6	0.017
Reaching up over head (%)	15.3	11.8	0.014
Grasp/holding small objects (%)	14.8	13.2	0.260
Going out to movies, events (%)	15.8	10.5	< 0.001
Attending social event (%)	11.4	6.8	< 0.001
Leisure activity at home (%)	5.1	2.8	0.004
Functional limitations^c^ ^,^ ^d^	2.0 (0.0, 6.0)	1.0 (0.0, 5.0)	< 0.001
Functional limitations severity score^e^	4.50 (4.15, 4.86)	3.10 (2.84, 3.36)	< 0.001
Poor general health (%)	36.5	49.2	< 0.001
Poor physical health (%)	18.4	13.9	< 0.001
Poor mental health (%)	12.8	9.9	0.018
Activity limitation (%)	10.5	6.9	< 0.001

*Note:* All values are weighted mean (95% CI) or weighted % unless otherwise stated.

^a^ dichotomised measure: a report of some or much difficulty or unable to do the activity was assigned a score of 1, while reporting no difficulty was assigned a score of 0.

Abbreviation: IQR‐interquartile range.

^b^ *P*‐value for differences between nonconsumers and consumers based on *F*‐test (continuous variable) or design‐based *F*‐test (categorical variables).

^c^ Values are median.

^d^ Functional limitations are based on the 19 NHANES questionnaire items, with a score range from 0 to 19.

^e^ Functional limitation severity score is a composite measure that multiplies the number of limitations (0–19) by the severity of each limitation (0–3), yielding a score range from 0 to 54.

Associations between nut consumption categories (nonconsumers (0 g/day) and nut consumers (> 0 g/day)) and the number and severity of reported functional limitations are presented in Table 3. Nut consumption (> 0 g/day) was associated with significantly fewer and lower severity of functional limitations compared to no consumption. To further investigate the potential dose at which the effect occurs, we examined the association between nut consumption categories (nonconsumers, consumers with intake below the median (< 6.9 g/day) and those with intake at or above the median (≥ 6.9 g/day)) and the number and severity of reported functional limitations; results are shown in Table 4. Compared with no consumption, an intake of nuts equal to or greater than the median in nut consumers (6.9 g/day) was associated with fewer functional limitations and a significantly lower severity of functional limitations score.

**TABLE 3:** Association of nut consumption categories (nonconsumers and nut consumers (≥ 0 g/day)) with the number, severity of reported functional limitations and health‐related quality of life among adults aged ≥ 60 years, NHANES, 2003–2012.

Outcome measure	Consumers (> 0 g/day)	
Functional limitations	Beta (95% CI)^a^	*P*‐value
Number of limitations^a^	−0.12 (−0.24, −0.01)	0.048
Severity score^b^	−0.67 (−1.11, −0.23)	0.004
**Health‐related quality of life** ^**d**^	**OR (95% CI)** ^**c**^	***P*** **‐value**
Poor general health^c^	0.84 (0.96, 1.41)	0.123
Poor physical health^c^	0.81 (0.68, 0.98)	0.027
Poor mental health^c^	0.90 (0.70,1.16)	0.395
Activity limitations	0.72 (0.55, 0.95)	0.022

*Note:* Exposure variables are consumers vs. nonconsumers, with nonconsumers as the reference group.

^a^ Values are negative binomial regression coefficients and 95% CI, adjusted for age, sex, BMI, ethnicity, household size, education status, smoking status, history of CVD, history of T2DM, history of arthritis, physical activity, and HEI‐2020.

^b^ Values are linear regression coefficients and 95% CI, adjusted for age, sex, BMI, ethnicity, household size, education status, smoking status, history of CVD, history of T2DM, history of arthritis, physical activity and HEI‐2020.

^c^ Data presented are odds ratios (OR) and 95% CI, adjusted for age, sex, BMI, ethnicity, household size, education status, smoking status, history of CVD, history of T2DM, history of arthritis, physical activity and HEI‐2020.

^d^ Each HRQOL‐4 component variable was categorised into good versus poor categories: general health was classified as good (good, very good or excellent health) versus poor (poor or fair), physical and mental health as good (< 14 days/month) versus poor (≥ 14 days/month) and activity limitation as good (< 14 days/month) versus poor (≤ 14 days/month) [27, 28].

**TABLE 4:** Association between categories of nut consumption with the number, severity of reported functional limitations and health‐related quality of life among adults aged ≥ 60 years, NHANES, 2003–2012.

Outcome measure	< Median^a^		≥ Median^a^	
Functional limitations	Beta (95% CI)	*P*‐value	Beta (95% CI)	*P*‐value
Number of limitations^b^	−0.05 (−0.21, 0.09)	0.464	−0.19 (−0.32, −0.06)	0.004
Severity score^c^	−0.52 (−1.06, −0.02)	0.061	−0.84 (−1.30, −0.37)	0.001
**Health‐related quality** **of life**	**OR (95% CI)**	***P*** **‐value**	**OR (95% CI)** ^**c**^	***P*** **‐value**
Poor general health^c^	0.98 (0.83, 1.27)	0.803	0.76 (0.60, 0.906)	0.023
Poor physical health^c^	0.88 (0.69, 1.11)	0.280	0.74 (0.57, 0.97)	0.027
Poor mental health^c^	0.94 (0.71, 1.26)	0.711	0.84 (0.61,1.16)	0.291
Activity limitations^c^	0.83 (0.57, 1.18)	0.295	0.61 (0.46, 0.82)	0.001

*Note:* Exposure variables are nonconsumers, consumers with intake < 6.9 g/day and consumers with intake ≥ 6.9 g/day, with nonconsumers as the reference group.

^a^ Median intake among nuts consumers (men) = 6.9 g/day.

^b^ Values are negative binomial regression coefficients and 95% CI, adjusted for age, sex, BMI, ethnicity, household size, education status, smoking status, history of CVD, history of T2DM, history of arthritis, physical activity and HEI‐2020.

^c^ Values are linear regression coefficients and 95% CI, adjusted for age, sex, BMI, ethnicity, household size, education status, smoking status, history of CVD, history of T2DM, history of arthritis, physical activity and HEI‐2020.

^d^ Data presented are odds ratios (OR) and 95% CI, adjusted for age, sex, BMI, ethnicity, household size, education status, smoking status, history of CVD, history of T2DM, history of arthritis, physical activity and HEI‐2020.

### 3.3 Nuts and Quality of Life

The associations between nut consumption (nonconsumers (0 g/day) and nut consumers (> 0 g/day)) and dichotomised HRQOL measures are presented in Table 3. Overall, nut consumption was significantly associated with lower odds of poor physical health and activity limitations. Nut consumers had an estimated 19% lower odds of experiencing poor physical health and 28% lower odds of activity limitations compared to nonconsumers. No significant association was observed between nut consumption and general or mental health. The association between nut consumption categories (nonconsumers, consumers with intake below the median (< 6.9 g/day)) and dichotomised HRQOL measures is shown in Table 4. Compared with no consumption, an intake of nuts equal to or greater than the median in nut consumers (6.9 g/day) was associated with lower odds of experiencing poor general health, poor physical health and activity limitations. No association was found between nuts and mental health.

## 4 Discussion

In this study of adults aged 60 years and over, we found that those who consumed nuts were more likely to experience fewer functional limitations and lower severity of functional limitations. Our analysis further showed that consuming nuts at or above the median intake among nut consumers (6.9 g/day), compared to no intake, was associated with fewer functional limitations and severity of these limitations. This intake level is comparable to those used in previous studies, which have reported that nut consumption at similar levels (e.g., ≥ 7 g/day) is associated with better overall diet quality, improved nutrient adequacy [31] and lower CVD risk factors [32].

Previous studies have reported an inverse association between nut consumption and functional limitations [12, 13]. For example, a prospective study from the Seniors‐ENRICA cohort reported a significantly lower risk of having issues with bending down or kneeling (HR = 0.59 [0.39–0.90]) and mobility in men (HR = 0.50 [0.29–0.90]) and a lower risk of impaired overall physical function in women (HR = 0.65 [0.48–0.87]) with higher nut consumption [13]. A recent prospective cohort study involving 9916 participants from the ASPREE Longitudinal Study of Older Persons found that daily nut consumption was associated with a 23% lower risk of reaching the disability‐free survival endpoint—a composite measure of survival without dementia or major physical disability over a mean follow‐up of 3.9 years (HR 0.77 [95% CI: 0.61–0.98]) compared to no or infrequent nut consumption [12]. These studies focused primarily on a limited range of physical function measures (i.e., ADLs) including bathing, dressing, toileting, transferring, continence and feeding [12], as well as specific tasks like bending or kneeling, carrying a shopping bag, climbing one flight of stairs and walking several city blocks [13].

In the present study, nearly half of those who did not consume nuts experienced limitations in stooping, crouching and kneeling. Similarly, a cohort study that included 1630 older adults aged ≥ 60 years showed that a diet high in nuts was associated with a lower likelihood of impairment in similar activities, including bending and kneeling [33]. A cross‐sectional study using NHANES data from 11 680 older adults showed that meeting the recommended protein intake (0.8 g/kg/day) as compared to not meeting the recommended protein intake is linked to improved self‐reported physical functioning, particularly in ADLs such as stooping, crouching or kneeling, standing or sitting for long periods, walking up 10 steps, preparing meals and walking for a quarter mile [34]. The relatively high energy and nutrient density of nuts makes them a potentially important inclusion in the diets of older adults to delay age‐related functional declines [13].

Our findings also indicate that nut consumers (> 0 g/day) are less likely to experience physically unhealthy and activity limitation days compared to nonconsumers. Further analysis showed that participants with nut intake equal to or greater than 6.9 g/day were also less likely to report poor general health, physically unhealthy days and activity limitation days relative to nonconsumers. Although previous studies have reported cognitive benefits from nut consumption, our study did not observe an association with mental health measures [9]. This may be because our mental health assessment focused specifically on recent experiences of self‐reported stress, depression and emotional problems, whereas previous studies [9, 35] examined longer term cognitive outcomes with objective measures, ranging from 8 weeks to 6.5 years.

A major strength of this study is its use of a large, nationally representative sample from NHANES. To better reflect habitual nut consumption, we only included participants who completed two 24‐h dietary recalls. Additionally, this study is the first to explore the relationship between nut consumption and HRQOL in older adults, providing support for future research into these areas. To the best of our knowledge, the association between nut consumption and functional limitations as a composite measure of 19 daily living activities, including instrumental tasks (e.g., managing finances, household chores and meal preparation) and social/leisure activities (e.g., attending events and engaging in hobbies) has not previously been reported. Maintaining independence in these activities is vital for maintaining one's quality of life, as limitations can lead to loss of independence, disability, poor quality of life and increased healthcare costs [36, 37]. The adjustment of diet quality using HEI‐2020 and physical activity allowed us to attribute the findings of this study to nut intake. However, several limitations must be acknowledged. First, due to the cross‐sectional nature of this study, only associations can be inferred, and causation cannot be established. Second, residual confounding may persist despite adjustments for potential confounding variables, including HEI‐2020 and physical activity. Third, we used self‐reported measurements of functional limitation, with the consequent potential for bias. Fourth, we were unable to adjust for certain pathologies (e.g., dementia and Parkinson's disease), which are known to contribute to both eating difficulties and other functional limitations, due to the unavailability of data on these in the NHANES dataset. Fifth, nuts are often consumed episodically, and two 24‐h recalls collected at NHANES may not adequately capture nut eating behaviours. This is a limitation, and future studies could consider estimating usual intake of nuts using various available modelling methods such as the NCI approach.

In conclusion, nut consumption was associated with fewer functional limitations and reduced severity of functional limitations in older adults. Furthermore, consuming nuts was associated with a reduced number of poor general, physically unhealthy and inactive days. Longitudinal and intervention studies are warranted to confirm the causal effect of nuts on self‐reported functional limitation and HRQOL.

## Ethics Statement

This study included data from NHANES 2003–2012, which obtained ethics approval from the National Center for Health Statistics (Protocol #98‐12, Protocol #2005‐06 and Protocol #2011–2017). All methods were performed in accordance with the relevant guidelines and regulations (Declaration of Helsinki), and all participants provided their informed consent.

## Conflicts of Interest

SYT, ESG, RMD and ENG have received research funding from the International Nut and Dried Fruit Council. SYT, ESG, RMD, ENG and JH have previously received research funding from the Peanut Institute (USA). SYT has also previously received research funding from the Almond Board of California.
